# Gender difference in effects of proprioceptive neuromuscular facilitation stretching on flexibility and stiffness of hamstring muscle

**DOI:** 10.3389/fphys.2022.918176

**Published:** 2022-07-22

**Authors:** Suiqing Yu, Lihua Lin, Hongying Liang, Ming Lin, Weixin Deng, Xinshu Zhan, Xihua Fu, Chunlong Liu

**Affiliations:** ^1^ Clinical College of Acupuncture, Moxibustion, and Rehabilitation, Guangzhou University of Chinese Medicine, Guangzhou, China; ^2^ Guangdong Industrial Injury Rehabilitation Hospital, Guangzhou, China; ^3^ Department of Infectious Diseases Unit, Panyu Central Hospital, Guangzhou, China

**Keywords:** PNF stretching, sex difference, muscle stiffness, flexibility, MyotonPRO, hamstring

## Abstract

**Objective:** This study investigated the acute effects of PNF stretching on hamstring flexibility and muscle stiffness of lower limbs between genders.

**Methods:** 15 male and 15 female university students without any injury histories on lower limbs in the past 3 months were included in this study were selected. All subjects were measured by MyotonPRO before and after stretching to determine the muscle stiffness of the biceps femoris muscle (BF), semitendinosus muscle (ST) of the hamstring and the medial gastrocnemius muscles (MG), lateral gastrocnemius muscles (LG), and the soleus (SOL) of the triceps surae muscles. Additionally, their flexibility was measured using the sit-and-reach test (the SR test) and passive hip range of motion (ROM). Differences based on time (pre-stretching vs. post-stretching) and sex (females vs. males) were assessed using 2 × 2 repeated measures AVONA.

**Results:** There was a significant decrease in the stiffness of the hamstring and triceps surae muscles after stretching (BF, MG, LG, and SOL: *p* < 0.001; ST: *p* = 0.003). The muscle stiffness of the hamstring and triceps surae muscles is larger in males than in females at all time points (*p* < 0.001). There was a significant increase in hip flexion angle and the SR test in males and females after PNF stretching (*p* < 0.001); However, there was no difference in the change in the muscle stiffness and the flexibility between genders (*p* > 0.05).

**Conclusion:** PNF stretching helped improve hamstring flexibility and decrease muscle stiffness. Stretching the hamstrings can also contribute to a decrease in the stiffness of the triceps surae muscles. The muscle stiffness of males before and after stretching is always greater than that of females. However, there was no difference in the change of improvement in stretching between genders.

## 1 Introduction

The hamstring muscle is a double joint muscle, which participates in the movement of hip extension and knee flexion at the same time. The degree of hamstring flexibility quality is the core key for individuals to promote their sports performance and maintain a good life pattern (Williams et al., 2019). According to the investigation, hamstring muscle strain is one of the most common injuries in sports (Kawai et al., 2021). To maintain muscle flexibility, enhance physical activity function and reduce the chance of injury, stretching is increasingly used in sports, fitness, and medical care. However, it was proved that stretching could not decrease the chance of injury in recent studies (Kim et al., 2018), and there is little evidence to support a relationship between increased flexibility and reduced incidence of injury (Van Mechelen et al., 1996; Thacker et al., 2004). In addition to not reducing the risk of sports injuries, it is generally accepted that stretching can effectively enhance flexibility, increase muscle strain points (Peck et al., 2014), and improve physical activity function (Oliveira et al., 2018). Flexibility is not only related to the capacity of athletes to complete technical movement, but also a vital factor affecting athletes’ performance.

Common stretching methods used to improve flexibility include static stretching, dynamic stretching, and PNF stretching (Gao et al., 2019). Each stretching method has its characteristics. However, some studies believe that static stretching and PNF stretching are superior to dynamic stretching in improving flexibility (Wong et al., 2011) and are one of the best methods to improve flexibility (Nakamura et al., 2021). But there has been debate about whether static stretching can harm athletic performance (Stafilidis and Tilp, 2015; Song et al., 2018). Although a lot of people debate whether there is a difference between static stretching and PNF stretching, many studies suggest that PNF stretching improve function and relieving pain more than static stretching (Tansu et al., 2019; Mani et al., 2021), and was more effective in improving flexibility of hip, shoulder and back (Wicke et al., 2014). The American College of Sports Medicine reports that PNF stretching is one of the most effective ways of stretching. Therefore, PNF stretching was selected for study in this experiment. PNF stretching is a special maneuver in stretching motion and decreasing muscle stiffness (Konrad et al., 2017). The basic principle is to stimulate as quite a few receptors as possible in the activity according to the physiological characteristics of neuromuscular, to enhance muscle activity and promote the realization of functional movement. It is characterized by first allowing strong muscle contraction to induce reflex self-inhibition, and subsequently using extension exercise to relax the muscle after the muscle relaxes due to reflex action. This allows the muscles to gain a range of motion. PNF stretching can be used either in the warm-up activity of training, in the relaxation phase after training, otherwise in motor rehabilitation to restore joint mobility.

It is well known that the connective tissues of males and females are physiologically distinct (Kjaer and Hansen, 2008). For instance, the muscle elasticity was different between males and females (Winter and Brookes, 1991a). Females have inherently greater compliance by comparison to males (Bryant et al., 2008). Although hamstring muscle strain may be multifactorial, the previously reported incidence of it is higher in males than in females. It is widely assumed that the same interventions in males and females lead to the same results. However, some studies have found that gender may cause differences in outcomes across interventions. As an example, Da Shilva et al. (2021) found a gender-dependent effect when skin temperature changes in response to exercise, In addition, the peak knee extension moment is greater in males than in females, and hamstring flexibility leads to different mechanical profiles in males and females (Williams and Welch, 2015). Since tendon stiffness and muscle volume are greater in males than in females, there may be structural differences between the sexes. If a fixed force is applied to a more pliable tendon, it will stretch further and therefore experience greater strain than a stiffer tendon. This in itself may affect the structural properties of the tendon to varying degrees during acute loading. The addition of a stretch might further exacerbate this sex difference. In summary, we hypothesize that acute stretching has different effects on tendon structure between the sexes. These proposed differential effects may have implications in both exercise and clinical settings.

Due to certain factors (e.g., difficulty in recruiting volunteers and the disparity between males and females ratios in the region), many studies have used the effect of stretching on a single-gender. However, we cannot speculate whether there is an effect of gender on the effect of stretching and whether the same intervention has different effects on gender constructs. Understanding the acute effect of PNF stretching on the architecture and mechanical properties of the hamstring muscle between genders has been a topic of interest among clinicians and researchers. Therefore, the purpose of the present investigation was to compare the acute effects of PNF stretching on flexibility and muscle stiffness in genders.

In clinical practice, for the interventions with the main purpose of reducing soft tissue stiffness and relaxing muscles, objective quantification of passive muscle stiffness of independent muscles is helpful to clarify its efficacy and is of great significance to both clinicians and scientific researchers. Our previous studies demonstrated that MyotonPRO is a valid and reliable tool to estimate the stiffness of muscles (Huang et al., 2018). It is well-known that fascia is continuous. When the fascia of a certain site is actively or passively pulled, the surrounding fascial chain will also be correspondingly changed. Due to the influence of anatomy and biomechanics, we can feel that the triceps surae muscle is also in an elongated state when the hamstring muscle is stretched. However, most studies are limited to investigating the structural and mechanical changes of the hamstring muscle itself, and few studies have reported changes in surrounding muscles, such as the triceps surae muscle. To this end, in addition to the hamstrings, we also measured muscle stiffness of the triceps surae muscle to understand fascial continuity more. In this study, the MyotonPRO was used to measure the stiffness of the hamstring and the triceps surae muscle before and after stretching. The sit-and-reach test (the SR test) and range of motion (ROM) (Palmer and Thiele, 2019) were used to observe the changes in the flexibility of the hamstrings, providing a more comprehensive understanding of the physiological characteristics of the independent muscle as well as a continuous muscular fascial chain. We hypothesized that after PNF stretching both groups of subjects showed increased flexibility after hamstring stretching and a decrease in muscle stiffness in both hamstrings and triceps calves, and that gender might affect the effect of stretching.

The objectives of this study were as follows: 1) to investigate the acute effects of PNF stretching on the muscle stiffness of the biceps femoris muscle (BF), semitendinosus muscle (ST) of the hamstring, and the medial gastrocnemius muscles (MG), lateral gastrocnemius muscles (LG) and soleus (SOL) of triceps surae muscles. 2) to investigate the acute effects of PNF stretching on the hamstring and lumbar flexibility. 3) to investigate any differences in the percentage change between genders caused by stretching.

## 2 Materials and methods

### 2.1 Ethics statement

The study was approved by the Human Subjects Ethics committee of the Clinical Medical College of Acupuncture, Moxibustion, and Rehabilitation, and was abided by the principles of the Declaration of Helsinki. Before the experiment, all subjects were informed of the experimental purpose, experimental procedures, rights of volunteers, and safety in the form of a written agreement by the researchers, and all signed informed consent.

### 2.2 Participants

Thirty healthy college students [15 males and 15 females; males: age: 21.13 ± 0.35 years; height: 1.73 ± 0.04 m; weight: 61.33 ± 5.97 kg; body mass index (BMI): 20.26 ± 1.73 kg/m^2^; females: age: 21.27 ± 0.80 years; height: 1.59 ± 0.05 m; weight: 51.31 ± 7.42 kg; body mass index (BMI): 20.30 ± 2.53 kg/m^2^] from Guangzhou University of Traditional Chinese Medicine were selected as the study subjects. The inclusion criteria were that all subjects were healthy right sharpshooters and could follow the instructions of the operator. The exclusion criteria were as follows: ①Subjects performed strenuous exercise within 48 h before the experiment. ② Injuries in the lower limb and history of low-back pain in the past 6 months. ③ History of neuromuscular disease, joint disease, or lumbar spine surgery. ④ Subjects had a BMI greater than 23.9 or lower than 18.5 kg/m^2^.

### 2.3 Experimental setup and protocol

This study was a self-controlled trial based on healthy subjects. The SR test, ROM of the hip joint, and muscle stiffness of the lower limbs were measured before (pre) and immediately after (post) stretching. The stiffness of the biceps femoris (BF), semitendinosus (ST), medial gastrocnemius (MG), lateral gastrocnemius (LG), and soleus (SOL) muscles of the dominant leg was measured. To reduce experimental error, all testing processes were carried out in the same location. The room temperature was kept at 25°C by air conditioning. And care was taken to ensure that all participants received the same instructions and verbal encouragement throughout all exercises and tests.

### 2.4 Measurements

#### 2.4.1 ROM examination of hip joint

**FIGURE 1:**
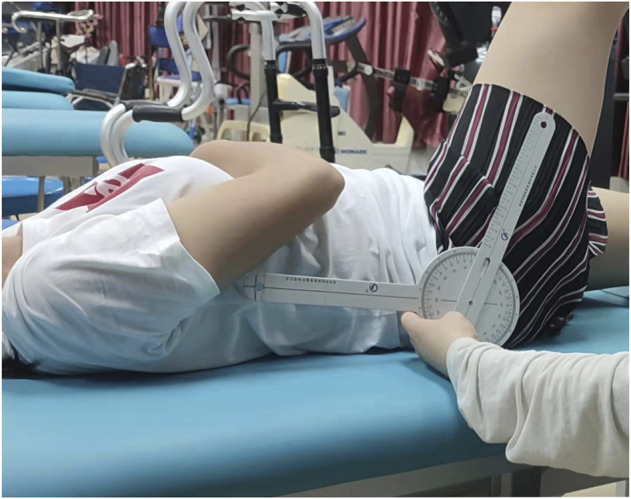
ROM examination of hip joint.

A manual goniometer was used to measure the hip joint angle (Sammons Preston, Royan, Canada). To determine hip mobility, the knee joint was set to 0°. The subject was instructed to gradually perform passive dorsiflexion until their maximum dorsiflexion angle was reached. ROM was measured three times in a row, at 5-s intervals. Following data analysis, the mean values were used (Figure 1).

#### 2.4.2 Measurement of SR test

**FIGURE 2:**
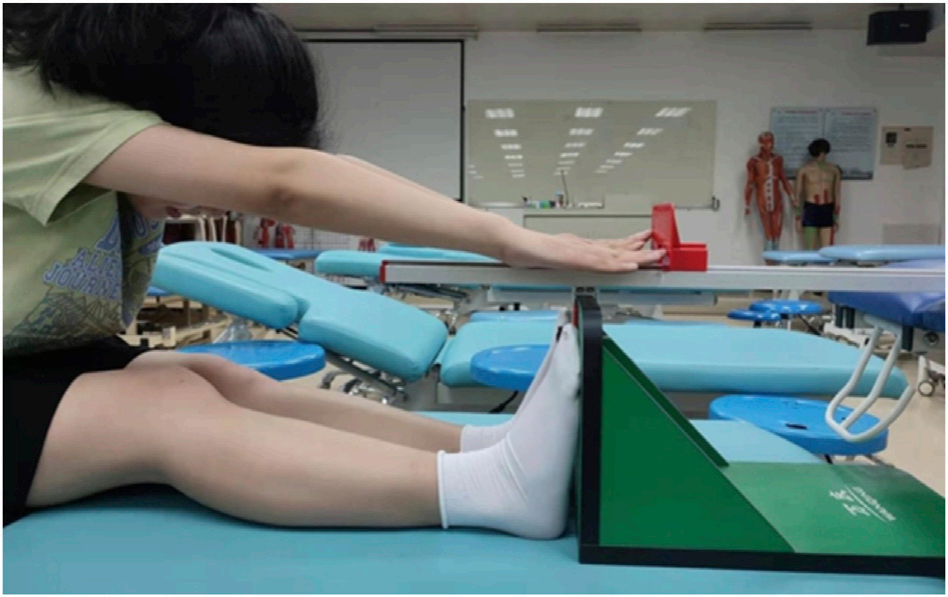
Measurement of the SR test.

The lumbar and hamstring flexibility was assessed using the standard SR test (Manzi et al., 2020). Participants were instructed to sit with their knees extended and their feet on the test box. The participants were then instructed to reach forward slowly along the top of the box and hold this position for 2 s while keeping their knees fully extended. Participants were graded based on the distance between their fingertips and the box (Figure 2).

#### 2.4.3 Measurement of muscle stiffness using MyotonPRO

A hand-held apparatus was used to evaluate the stiffness of the biceps femoris (BF), semitendinosus (ST), medial gastrocnemius (MG), lateral gastrocnemius (LG), and soleus (SOL) muscles (Model: MyotonPRO, produced by MyotonPRO AS in Estonia). The participants were positioned in a prone position, with their hands flat on both sides of their bodies and their feet naturally hung on the bed’s edge. The following are the muscles that were discovered and located. ① BF: 50% of the distance from the isocenter to the lateral epicondyle of the femur; ② ST: 50% of the distance from the isocenter to the medial epicondyle of the femur (Pincivero et al., 2000); ③ MG: 70% of the distance between the lateral leg bone and the medial popliteal fossa; ④ LG: two-thirds of the distance between the root tubercle and the fibular head; ⑤ SOL: two-thirds of the distance between the medial femoral condyle and the tip of the medial leg bone.

**FIGURE 3:**
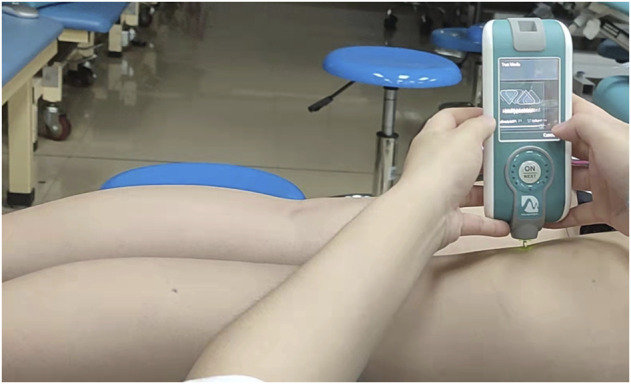
Measurement of muscle stiffness using MyotonPRO.

The following are the specific measurement steps. 1) The subjects are placed in the prone position after 5 min of total body relaxation; 2) To complete the measurement of muscle stiffness, the assessor held the MyotonPRO probe perpendicular to the mark and gently touched the MyotonPRO until the probe showed a green line (Figure 3). The order of muscle stiffness measurements in the different states was randomized, with each muscle measured three times, with one-minute rest between each measurement, and then averaged for analysis.

### 2.5 Stretching protocol

**FIGURE 4:**
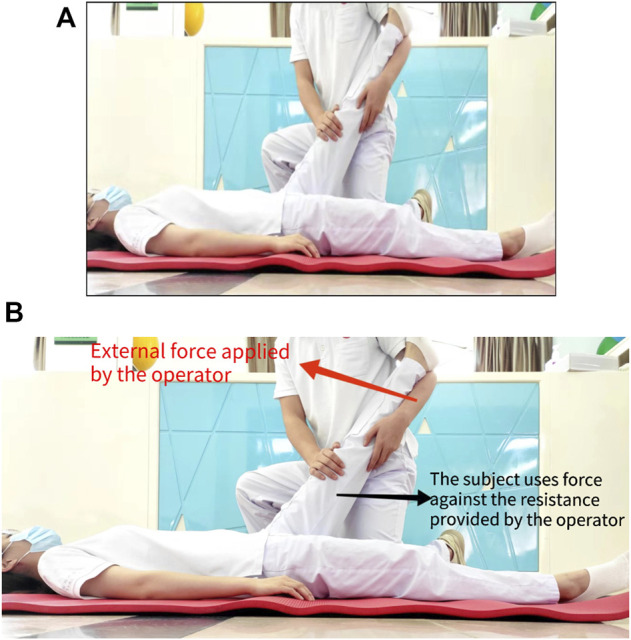
**(A)** Diagram of passively stretch. **(B)** Diagram of the subject contract the hamstring isometric.

The subject was placed in the supine position. Firstly, stretch the hamstring passively until the subject feels mildly uncomfortable and hold for 10 s (Figure 4A); Secondly, the subject against the operator forcefully contracted the hamstring equidistant for 6 s while keeping the leg position unchanged (Figure 4B); Thirdly, release it, and then passively stretch for another 30 s (Figure 4A). Three sets of PNF stretches were performed, with a 30-s rest period between each set.

## 3 Statistical analysis

The most similar previous study found that after stretching, muscle stiffness measurements were both reliable and statistically accurate in a sample of 10 male and 11 female health participants (Simpson et al., 2018). Based on his research, the study enrolled 15 males and 15 females expected to show sufficient reliability estimation accuracy and provide sufficient statistical ability to distinguish between muscle states before and after stretching.

All statistical analyses were performed using SPSS (version 25.0 Chicago, IL), and data were presented as mean ± standard deviation. The Shapiro-Wilk test was used to analyze the positivity of the data. An independent sample *t*-test was used to compare the general data. 2 × 2 repeated measures ANOVA [time (pre-stretching vs. post-stretching) X sex (females vs. males)] was used to ascertain if hamstring flexibility (the SR test and ROM) and muscle stiffness of hamstring and triceps surae muscles were altered for males and females after an acute stretch. The significance level for the tests was set at *p* < 0.05.

## 4 Results

### 4.1 Changes in the SR test

**FIGURE 5:**
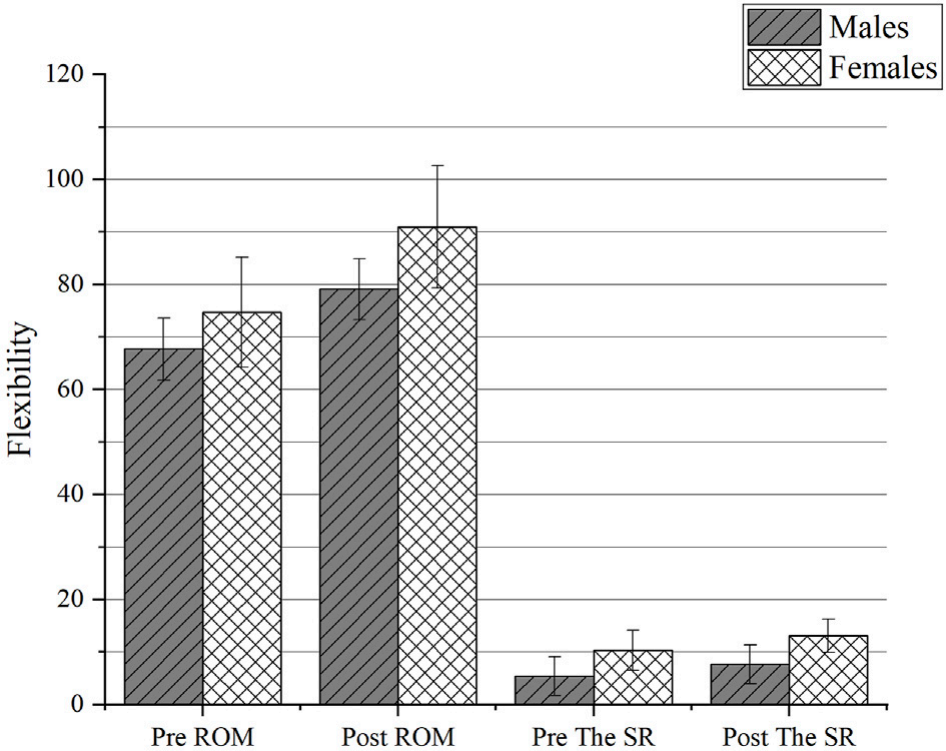
The change of flexibility between genders. Abbreviations: the SR test, the sit-and-reach test; ROM, range of motion; Pre, pre-stretching; Post, post-stretching.

The performance of the SR test in males and females before and after PNF stretching was shown in Tables 1, 2 and Figure 5. The difference in the SR test before and after stretching was statistically significant (F = 24.268, *p* < 0.001). There was no interaction between gender and time, and the scores of the SR test of genders did not vary with stretching time. There was no difference in the improvement in the SR test between males and females groups (F = 0.172, *p* = 0.681). A significant effect for sex existed throughout the study, with females outperforming males in terms of flexibility (F = 18.487, *p* < 0.001).

**TABLE 1:** Comparison of hamstring flexibility and muscle stiffness between genders.

	Males				Females			
	Pre-stretching (kPa)	Post-stretching (kPa)	Percentage change	*p* values	Pre-stretching (kPa)	Post-stretching (kPa)	Percentage change	*p* values
ROM	67.7 ± 5.9	79.1 ± 5.8	−16.84%	<0.001	74.7 ± 10.5	91.0 ± 11.7	−21.82%	<0.001
The SR test	5.4 ± 3.7	7.7 ± 3.7	−42.59%	0.003	10.4 ± 3.8	13.1 ± 3.2	−25.96%	0.004
BF	298.9 ± 9.7	286.2 ± 6.4	4.25%	<0.001	221.3 ± 7.5	207.6 ± 10.6	6.19%	<0.001
ST	291.4 ± 8.3	284.0 ± 11.0	2.09%	0.036	216.2 ± 12.2	210.7 ± 9.0	1.02%	0.029
MG	285.1 ± 5.2	271.9 ± 2.0	4.63%	<0.001	253.7 ± 9.5	247.0 ± 4.4	2.64%	0.015
LG	308.9 ± 7.8	293.0 ± 2.4	5.15%	<0.001	263.1 ± 11.6	254.8 ± 9.0	3.15%	0.013
SOL	416.1 ± 9.1	401.3 ± 8.5	3.56%	<0.001	371.5 ± 8.0	349.6 ± 8.5	5.90%	<0.001

Values are means ± SD. Percentage change = (posttest values − pretest values) ÷ pretest values × 100%. Abbreviations: ROM, range of motion; The SR test, the sit-and-reach test; BF, biceps femoris muscle; ST, semitendinosus muscle; MG, medial gastrocnemius muscles; LG, lateral gastrocnemius muscles; SOL, soleus.

**TABLE 2:** Effects of time, gender, and their interactions.

	Time effect		Gender effect		Interaction between gender and time	
	F values	*p* values	F values	*p* values	F values	*p* values
ROM	68.444	<0.001	11.645	0.002	2.118	0.157
The SR test	24.268	<0.001	18.487	<0.001	0.172	0.681
BF	61.620	<0.001	835.446	<0.001	0.084	0.774
ST	10.888	0.003	540.553	<0.001	0.246	0.624
MG	32.961	<0.001	157.168	<0.001	3.662	0.066
LG	34.511	<0.001	216.664	<0.001	3.464	0.073
SOL	100.427	<0.001	362.227	<0.001	3.787	0.062

Values are means ± SD. Abbreviations: ROM, range of motion; The SR test, the sit-and-reach test; BF, biceps femoris muscle; ST, semitendinosus muscle; MG, medial gastrocnemius muscles; LG, lateral gastrocnemius muscles; SOL, soleus.

### 4.2 Changes in ROM

Tables 1, 2 and Figure 5 illustrate the ROM performance of males and females before and after PNF stretching. The difference in mean ROM before and after stretching was statistically significant (F = 68.444, *p* < 0.001). There was no relationship between gender and time, and neither gender’s mean ROM changed with stretching duration. The improvement in ROM did not differ between the male and female groups (F = 2.118, *p* = 0.157). A significant effect for sex existed throughout the study, with females demonstrating greater hip ROM than males did at each time point (F = 11.645, *p* = 0.002).

### 4.3 Changes in stiffness in different regions

**FIGURE 6:**
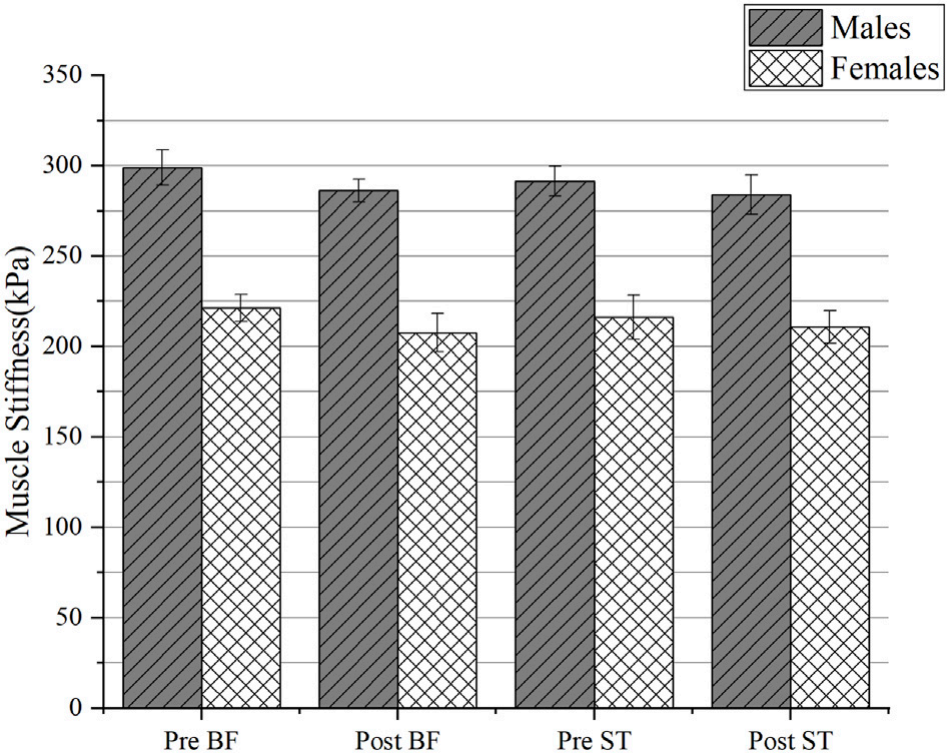
The change of muscle stiffness of triceps calf muscles between genders. Abbreviations: MG, medial gastrocnemius muscles; LG, lateral gastrocnemius muscles; SOL, soleus; Pre, pre-stretching; Post, post-stretching.

**FIGURE 7:**
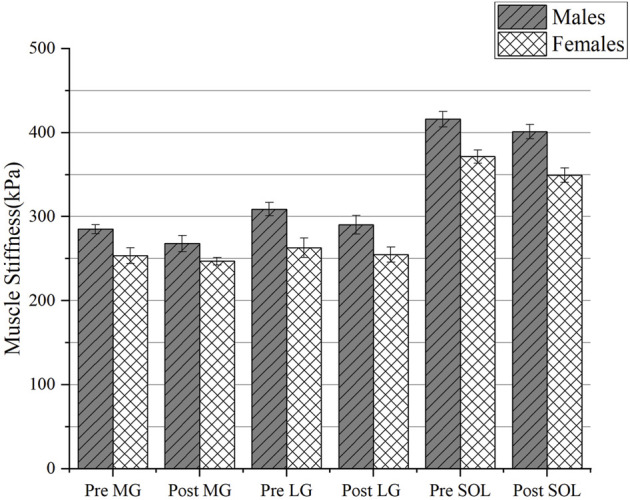
The change of muscle stiffness of hamstring muscles between genders. Abbreviations: BF, biceps femoris muscle; ST, semitendinosus muscle; Pre, pre-stretching; Post, post-stretching.

The changes in muscle stiffness in different regions of males and females before and after PNF stretching were shown in Tables 1, 2 and Figures 6, 7. There was no interaction between gender and time, and the mean muscle stiffness of genders did not change with stretching time. There was no difference in the decrease of muscle stiffness between males and females groups (BF: F = 0.084, *p* = 0.774; ST: F = 0.246, *p* = 0.624; MG: F = 3.662, *p* = 0.066; LG: F = 3.464, *p* = 0.073; SOL: F = 3.787, *p* = 0.062). There was statistically significant difference in mean muscle stiffness before and after stretching (BF: F = 61.620, *p* < 0.001; ST: F = 10.888, *p* = 0.003; MG: F = 32.961, *p* < 0.001; LG: F = 34.511, *p* < 0.001; SOL: F = 100.427, *p* < 0.001). A significant effect for sex existed throughout the study. Males’ muscle stiffness was significantly higher than females’ at all time points (BF: F = 835.446, *p* < 0.001; ST: F = 540.553, *p* < 0.001; MG: F = 157.168, *p* < 0.001; LG: F = 216.664, *p* < 0.001; SOL: F = 362.227, *p* < 0.001).

## 5 Discussion

Our research looked at whether PNF stretching of the hamstrings improved hamstring flexibility and muscle stiffness and whether it affected the adjacent calf triceps. We also investigate whether gender influences the effect of PNF stretching to better understand the structural and physiological differences between males and females and to solve the uneven gender recruitment of volunteers that frequently occurs during clinical trials. We discovered that PNF stretching not only improves hamstring muscle flexibility but also reduces hamstring muscle stiffness and surrounding muscle stiffness. According to the findings of this study, females are more flexible than males. The stiffness of female muscles is less than that of males. Acute stretching, on the other hand, did not affect the relationship between these components in either males or females.

### 5.1 Acute effects on flexibility between genders

Flexibility is an important physiological component of physical fitness, and reduced flexibility can lead to inefficiencies in the workplace and is a risk factor for low back pain. Several studies had investigated the stretching effects on muscle properties in males and females. It is accepted that females are considered to be more flexible than males (Tsolakis and Bogdanis, 2012; McPherson et al., 2020; Höög and Andersson, 2021), which supports our finding. The SR test and ROM were found to increase in both males and females after an acute passive stretch in the above test.

The previous study suggested that the effect of stretching on ROM may be related to a modification in stretch tolerance (Marek et al., 2005). Concerning the mechanism of altered stretch tolerance, afferent inputs from muscles and joints during stretch may suppress signals from nociceptive fibers, which may increase pain thresholds (Yu and Shin, 2019). In addition, sensory afferents may affect the release of enkephalin from interneurons, which may help to reduce pain transmission in the dorsal horn during stretching, thereby increasing the pain threshold. The analgesic effect achieved by increasing the pain threshold may have altered the tensile tolerance, resulting in a range of motion. In addition, another mechanism regarding the flexibility that PNF stretching can improve is reflexive inhibition (Cayco et al., 2019). Let the muscles contract strongly to induce reflexive self-inhibition, and then use stretching exercises to relax the muscles after they have relaxed due to the reflexive effect, thus obtaining a greater range of motion. However, there was no significant difference in the change amount between genders, which is in agreement with the previous study (Decoster et al., 2004). GIORGOS P shows that both static stretching and dynamic stretching improved flexibility performance with no gender interaction being found (Paradisis et al., 2014). Tsolakis and Bogdanis (2012) found that at all time points, females had greater ROM than males, but the pattern of change in hip flexibility was not different between genders. These conclusions are consistent with ours (Cipriani et al., 2012). However, only two studies found that stretching improved flexibility in females more than in males. Katherine M. examined musculotendinous stiffness and ankle ROM in males and females after an acute bout of passive stretching and found that ROM increased from pre to post-stretching for the females (*p* < 0.001), but not the males (Hoge et al., 2010). However, the females in their study were all tested during menses. Estrogen has previously been shown to inhibit collagen synthesis and thus affect tendon tissue quality, hormonal differences between genders could affect tendonous tissue (Kjaer et al., 2006). To avoid this, our volunteers were elected not during the menstrual period. Rayamajhi’s study found that PNF maintaining-relaxation extension had a positive effect on improving rectus femoris flexibility, and this effect was more obvious in females. The reason their study differed from ours may be that their target muscle group was the rectus femoris, while ours was the hamstring. And we do not stretch as often as they do, we performed three sets of PNF stretching, while they stretched six times for a week. Notably, there was no change in muscle stiffness between days 0 and 3 in both males and females in their study (Rayamajhi et al., 2014).

### 5.2 Acute effects on muscle stiffness between genders

In our study, we have found that the stiffness of the hamstring decreased significantly after PNF stretching, which is in agreement with the results reported by Konrad et al. (2015), and supports previous findings (Hill et al., 2017; Lempke et al., 2018). While the detailed mechanism underlying the reduction in muscle stiffness after PNFS is unknown, the acute effects of PNFS on the properties of intramuscular connective tissue such as endomysium, perimysium, and apparent may also contribute to the reduction in muscle stiffness after PNF stretching. The possible reason for the decrease in muscle stiffness after acute stretching may be that the collagen fibers in the unstressed tendon straighten wavy when stretched (Stromberg and Wiederhielm, 1969). One can speculate that the straightening process of fibers becomes easier due to the customary stretching. An alternative explanation was proposed by McNair et al. (2001) when studying circular motion. They speculated that polysaccharides and water were redistributed within the collagen framework, which resulted in a reduction in stiffness (McNair et al., 2001). Moreover, we also found the stiffness of the triceps surae decreased. The calf triceps is located on the posterior side of the calf muscles and consist of the soleus and gastrocnemius. The gastrocnemius is a double joint muscle, starting at the medial and lateral femoral condyles and ending at the heel tuberosity, and functions as a knee flexor and ankle plantar flexor. Due to the morphological continuity between the gastrocnemius and hamstrings, stretching the thigh causes displacement of the soft tissue on the dorsal side of the calf. The deep fascia of the popliteal fossa connects the hamstrings and gastrocnemius. In addition, Wilke and Tenberg (2020) found that passive ankle dorsal extension resulted in significant displacement of the semimembranosus muscle and its fascial band, suggesting that mechanical force can be transmitted from the ankle joint to the dorsal thigh, affecting the parallel muscles contained in the dorsal thigh fascial band. The presence of myofascial chains that muscles do not exist functionally and structurally in isolation. This is consistent with the conclusion that muscle stiffness changes in the triceps surae muscle in this experiment.

Another major finding in this experiment was the difference in muscle stiffness between genders. According to the previous studies, the muscle stiffness in males is greater than in females (Winter and Brookes, 1991; McPherson et al., 2020). Which were in agreement with the results we had in our study. It was reported that differences in hamstring tendon stiffness (MTS) by gender lead to differences in electromechanical delay and rate of force production. Sex differences in these characteristics may lead to a higher risk of ACL injury in females (Blackburn et al., 2009). Furthermore, we discovered an intriguing phenomenon in which the muscle stiffness in the hamstring (BF, ST) and triceps calf muscles in females is approximately 70%–80% of that of males (including MG, LG, SOL) which supports previous findings (Chino and Takahashi, 2016). In our study, we found no significant difference in the change amount of PNF stretching between genders. This is consistent with the conclusions of others (Simpson et al., 2018). The corresponding theory is that there are no systematic differences in fiber type due to sex (Jeon et al., 2019). However, Burgess et al. (2009) performed passive dorsiflexion stretching for 5 min and found that muscle stiffness of the medial gastrocnemius tendon decreased more in females than in males after stretching. The reason for the different results might be that they applied static stretching for 5 min, while PNF stretching was adopted three times in our experiment.

Though the effects of sex on stretching were controversial, the PNF stretching could be recommended as part of the warm-up, particularly for sports that require high flexibility (e.g., gymnastics, ballet, diving). PNF stretching has a good effect on improving overly strengthened muscles, and could also improve sports performance. In addition, during the stretching process, the coach and the member need to cooperate, so could better improve the interactivity and fun of the course. However, something has to be emphasized when doing PNF stretching. Due to the reduction of subjective pain sensation during the exercise, it is easy to cause too much stretching and aggravate the injury if care is not taken. In the practice process, we should focus our minds to ensure that the action posture is standardized, and control the amplitude of the stretching. In the active contraction phase of PNF stretching, the degree of force should be at 50% of MVC (Lim, 2018; Kay et al., 2020)and the maximum stretching amplitude in the stretching process should be the appearance of the muscle tautness and soreness instead of the appearance of pain (Cornelius and Hands, 1992). What is more, it is recommended that underage and poorly trained individuals do it sparingly.

MyotonPRO was able to effectively detect the change in stiffness of hamstrings as well as triceps calves before and after stretching, which helped us to understand the structural differences between genders and quantify the effect after PNF stretching. Based on this, the next step in future research is to use MyotonPRO to help optimize the ideal and most effective PNF protocol, such as duration, intensity, frequency, and repetitions.

### 5.3 Limitation

Only healthy subjects were included in this study, and the limitation of joint motion was not significant. Secondly, the influence of exercise history and other influencing factors on muscle stiffness is not considered enough, resulting in a certain experimental error. In addition, because each muscle in this study was only localized in one location for measurement and the sample size was small, it remains to be evaluated in a longitudinal study with a large sample. Further studies can look at changes in hamstring stiffness in pathological conditions and evaluate the efficacy of interventions.

## 6 Conclusion

The study indicated that PNF stretching improved hamstring flexibility. Both the SR test and ROM can be used as indicators to detect improvements in flexibility. Stretching the hamstrings reduced the stiffness of the hamstrings as well as the triceps calf muscles, confirming that the stretching effect is not limited to the local muscle being stretched. MyotonPRO is a reliable device for assessing muscle stiffness. Despite the structural differences between males and females, with males being much stiffer than females, there was no difference in the effect of gender on PNF stretching.

## Data Availability

The original contributions presented in the study are included in the article/Supplementary Material, further inquiries can be directed to the corresponding authors.
